# Classifying multiple types of hand motions using electrocorticography during intraoperative awake craniotomy and seizure monitoring processes—case studies

**DOI:** 10.3389/fnins.2015.00353

**Published:** 2015-10-01

**Authors:** Tao Xie, Dingguo Zhang, Zehan Wu, Liang Chen, Xiangyang Zhu

**Affiliations:** ^1^State Key Laboratory of Mechanical System and Vibration, Institute of Robotics, Shanghai Jiao Tong UniversityShanghai, China; ^2^Department of Neurosurgery, Huashan Hospital, Fudan UniversityShanghai, China

**Keywords:** electrocorticography (ECoG), brain-computer interface (BCI), hand movements, feature extraction, classification

## Abstract

In this work, some case studies were conducted to classify several kinds of hand motions from electrocorticography (ECoG) signals during intraoperative awake craniotomy & extraoperative seizure monitoring processes. Four subjects (P1, P2 with intractable epilepsy during seizure monitoring and P3, P4 with brain tumor during awake craniotomy) participated in the experiments. Subjects performed three types of hand motions (Grasp, Thumb-finger motion and Index-finger motion) contralateral to the motor cortex covered with ECoG electrodes. Two methods were used for signal processing. Method I: autoregressive (AR) model with burg method was applied to extract features, and additional waveform length (WL) feature has been considered, finally the linear discriminative analysis (LDA) was used as the classifier. Method II: stationary subspace analysis (SSA) was applied for data preprocessing, and the common spatial pattern (CSP) was used for feature extraction before LDA decoding process. Applying method I, the three-class accuracy of P1~P4 were 90.17, 96.00, 91.77, and 92.95% respectively. For method II, the three-class accuracy of P1~P4 were 72.00, 93.17, 95.22, and 90.36% respectively. This study verified the possibility of decoding multiple hand motion types during an awake craniotomy, which is the first step toward dexterous neuroprosthetic control during surgical implantation, in order to verify the optimal placement of electrodes. The accuracy during awake craniotomy was comparable to results during seizure monitoring. This study also indicated that ECoG was a promising approach for precise identification of eloquent cortex during awake craniotomy, and might form a promising BCI system that could benefit both patients and neurosurgeons.

## 1. Introduction

Electrical recordings from surface of brain cortex [i.e., electrocorticography (ECoG)], have been recognized as a promising signal resource not only for clinical application (e.g., eloquent cortex identification during awake craniotomy, Roland et al., 2010, or epileptic seizure localization) but also for brain-computer interface (BCI) research (Schalk and Leuthardt, 2011). Clinical and BCI research may benefit from each other. Clinical research gave the access to the valuable ECoG signal, and BCI research could enhance our understanding of the ECoG characteristic and expand its application.

For a pure BCI-purpose implantation, it would be highly necessary to demonstrate reliable neuroprosthetic control during surgical implantation (i.e., during awake craniotomy), in order to verify the optimal placement of electrodes (Fifer et al., 2014). Non-invasive methods of functional mapping (e.g., fMRI) could only be used for gross surgical planning, but intraoperative verification of control with ECoG would be extremely useful to refine the final implantation site. This would help to avoid the need for re-implantation because the patient was unable to control the neuroprosthetic.

Precise identification of eloquent cortex was a clinical necessity prior to surgical resections adjacent to motor cortex. ECoG signals could serve as a useful adjunct to cortical stimulation mapping during the intraoperative setting (Roland et al., 2010; Kamada et al., 2014). It also possessed the potential to increase the identification efficiency and resolution of the motor cortex (Leuthardt et al., 2007), and reduce the amount of necessary electrocortical stimulation during the awake craniotomy process. However, motion tasks within these studies were simply opening and closing the contralateral hand, and dexterous hand motion types may have the potential for more precise localization of eloquent cortex.

There was a general agreement that low-frequency (<25 Hz) components of the electroencephalography (EEG) decrease in amplitude after onset of movement over the contralateral sensorimotor cortex, and this finding was later found in subdural ECoG (Miller et al., 2007). Compared with EEG, additional increases of ECoG in the amplitude of higher frequency components (<35 Hz) were described in relation to movement (Crone et al., 1998; Miller et al., 2007), and those changes had a more localized spatial distribution on the surface of brain cortex than low-frequency changes. Signals at higher frequencies were demonstrated to carry more detailed information about hand movements (Schalk and Leuthardt, 2011), thereby providing more critical information which was hardly accessible with EEG-based BCI.

On the early stage, ECoG-based BCI focused on classifying simply movements of different body parts, such as hand, foot and tongue movements based on neural oscillatory dynamics (Graimann et al., 2003). Subsequently, predicting the kinematic and kinetic parameters of human hand and finger motions aroused much interest. Spatially distinct brain regions were specific to individual fingers (Miller et al., 2009), and the flexion time courses were highly specific to fingers (Kubánek et al., 2009). Individual finger flexion and movements were decoded with particularly high correlation coefficient (0.46 in Liang and Bougrain, 2012, 0.42 in Flamary and Rakotomamonjy, 2012), and multiple types of hand posture and grasps have been classified with “macro” (Pistohl et al., 2012; Chestek et al., 2013) and “micro” (Bleichner et al., 2014) electrode grids. Five isometric hand postures could be classified correctly with an average of 77.6% (20% chance) with three subjects (Chestek et al., 2013), and for four complex hand gestures, two participants achieved 97 and 74% (25% chance) classification accuracy respectively (Bleichner et al., 2014). However, all these studies were carried out after the implantation.

We here test the hypothesis that reliable identification of multiple types of hand motions can be realized on subjects under awake craniotomy, which is comparable to the decoding results of subjects during epileptic seizure monitoring. This is the first step toward the dexterous neuroprosthetic control during surgical implantation, in order to verify the optimal placement of electrodes that are implanted for pure BCI-purpose, and aimed for more precise localization of eloquent cortex during intraoperative surgical process.

Considering the clinical condition and current studies for multiple hand motion types decoding (Liang and Bougrain, 2012; Bleichner et al., 2014), three types of simple hand motions (i.e., grasp, thumb-finger motion and index-finger motion) were performed by four subjects (two brain tumor subjects undergoing an awake craniotomy, two epilepsy subjects during seizure monitoring, for one epilepsy subject in good condition, two more hand motion types, i.e., hand unfold and wrist motion, were performed). To further validate the decoding results, two methods were applied in this study. Method I: autoregressive (AR) model with burg method was applied to extract features, and additional waveform length (WL) feature has been considered, finally the linear discriminative analysis (LDA) was used as the classifier. Method II: stationary subspace analysis (SSA) was applied for data preprocessing, and the common spatial pattern (CSP) was used for feature extraction before LDA decoding process.

## 2. Methods

### 2.1. Subjects

**Figure 1:**
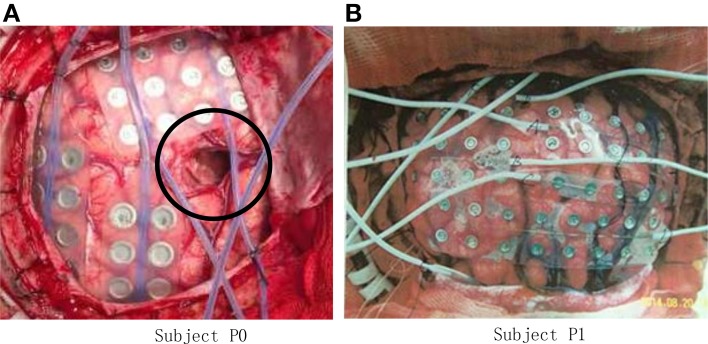
**Photo of ECoG grids placed on the brain cortex surface**. **(A)** Grid photo of Subject P0, the black circle indicates the resection cortex of brain tumor; **(B)** Grid photo of Subject P1.

The experiment was conducted on five right-handed human subjects. Subject P0 (Figure 1A) suffered from intractable epilepsy, and underwent temporary placement of subdural electrode arrays, however, he has already underwent resection of brain tumor before been diagnosed with epilepsy (shown within the black circle of Figure 1A), Hand motion information could not be decoding from ECoG signal of P0, thus Subject P0 was excluded and four subjects were considered in the study.

Subject P1 (male, 14 years old), P2 (male, 30) suffered from intractable epilepsy, and they underwent temporary placement of a subdural electrode array in order to localize the epileptic seizure focus and map brain function prior to surgical resection (Figure 1B). The epileptic focus was found to be left posterior frontal for P1 and right for P2. Platinum grid electrodes (4 mm electrode diameter and 1 cm inter-electrode distance, 87 contacts for P1, and 95 contacts for P2) were subdurally implanted above the region of corresponding hemisphere. The sites of electrode implantation were exclusively based on the requirements of the clinical evaluation.

Subject P3 (male, 60), P4 (female, 61) suffered with brain tumor, and they were undergoing an awake craniotomy for the treatment of tumors adjacent to motor and speech cortex. Platinum grid electrodes (4 mm electrode diameter and 1 cm inter-electroded,16 contacts for P3 and 52 contacts for P4) were subdurally implanted above the region of the left hemisphere for both of the subjects.

All the subjects were recruited from Huashan Hospital and were informed about the whole experiment process. This study was approved by the Ethics Committee of Huashan Hospital. All subjects signed the informed consent forms by themselves or legal guardians before participating in the experiments.

### 2.2. ECoG data recording

ECoG data of subjects P1, P3, P4 were recorded from the electrode grid connected to the Stellate eAMP 64 (Canada) system, and CEEGRAPH (128+ channal XL 2) was used on subject P2. The signals were amplified, bandpass filtered between 0.15~100 Hz, digitized at 250, 256, 200, and 2000 Hz for P1~P4, respectively.

### 2.3. Experimental design

#### 2.3.1. Setup for subjects P1, P2

**Figure 2:**
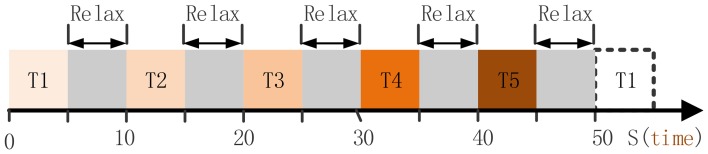
**The full run experiment of subject P1**. T1, hand unfold; T2, hand grasp; T3, thumb-finger motion; T4, wrist motion; T5, index-finger motion. Each trial lasts 5 s, thereby 50 s were needed for the full run.

Subject P1 was lying in a semi-recumbent position in a hospital bed. He was instructed to perform the given tasks with the hand contralateral to the implant in response to a sound clip. A total of 100 trials were performed by subject P1 in 20 runs. Each run contained 5 trials which indicated different hand motion types (Figure 2), i.e., hand unfold (T1), hand grasp (T2), thumb-finger motion (T3), wrist motion (T4), and index-finger motion (T5). For each trial, the subject repeated to do a given motion type for 5 s, e.g., repeating to perform hand grasp with normal speed, and then following a relaxation period with 5 s. Data were collected for a total period of about 20 min, which yielded an average of 20 trials for each hand motion type. During the data collecting process, any other movements including facial or head movements were avoided.

Subject P2 did the similar tasks as P1, and three hand motion types were executed : hand grasp, thumb-finger motion, index-finger motion. A total of 60 trials were performed in 20 runs, and thus yielded an average of 20 trials for each types.

#### 2.3.2. Setup for subjects P3, P4

**Figure 3:**
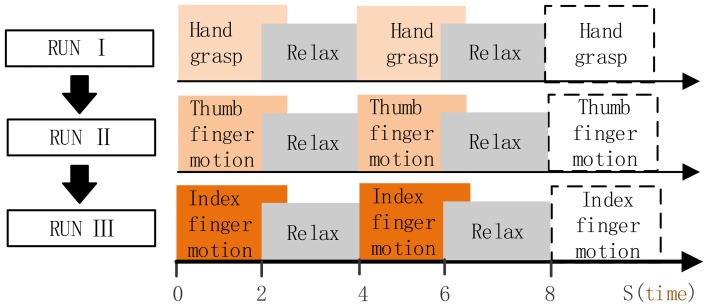
**Full run experiment of subject P3, P4**. RUN I, hand grasp; RUN II, thumb-finger motion; III, index-finger motion. Each run contained 20 trials, each trial lasts 4~6 s.

Subject was in the operating room, and was instructed to perform the given tasks with the hand contralateral to the implant in response to the voice commands. Three motion types were performed in separate run (Figure 3), i.e., run I for hand grasp, run II for thumb-finger motion and run III for index-finger motion. Twenty trials were performed for each run, and 60 trials during the whole experiment. For each trial, subject performed the given task just once instead of repetitively performing the task (as P1, P2), the execution time last for 2~3 s and then following a relaxation period with 2~3 s. Data were collected for a total period of about 5 min, which yielded an average of 20 trials for each motion type.

### 2.4. Offline analysis

Before decoding of hand motion types, the data during the awake craniotomy and seizure monitoring process was analyzed offline to investigate which electrodes and frequency ranges carry more task-relevant information. The raw signal was re-referenced to the common average by subtracting the mean value over all channels at each time point. The autoregressive model of order 60 with Burg method (Harris, 1967) was applied to estimate the power spectrum for each data segment. Logarithmic power of 1 Hz frequency bins from 1~100 Hz were used to calculated the *R*^2^-values (Sheikh et al., 2003) between all relevant combinations of two hand motions for each frequency bin at each electrode. Channels with high *R*^2^-values indicated more task-relevant information.

### 2.5. Algorithms for decoding hand motion types

To further validate the decoding results, two methods (Method I and Method II) were applied in this study.

#### 2.5.1. Method I: AR-LDA with WL feature

Before decoding of hand motions, the frequency ranges and electrodes that carry more task-relevant information need to be selected. The raw signal was re-referenced to the common average by subtracting the mean value over all channels at each time point. The autoregressive model of order 60 with Burg method (Harris, 1967) was applied to estimate the logarithmic power spectrum for each data segment. We calculated the *R*^2^-values (Sheikh et al., 2003) between all relevant combinations of two hand motions for each 10 Hz frequency bin at each electrode. To reduce dimensionality of the input space, 5 electrodes with 5 frequency ranges within each electrode were selected for classification according to the *R*^2^-values. Thus, 25 features (*AR* feature) were selected for a single trial.

In this study, we also considered another informative feature: Waveform Length feature. WL is a measure of signal complexity (Tkach et al., 2010), and has been proved to be a robust and efficient feature for electromyography (EMG) (Tkach et al., 2010) and EEG (Lotte, 2012) signals. The WL feature could be extracted from an ECoG signal as follows (Tkach et al., 2010):

(1) $$\begin{array}{c} wl=log(\sum_{i\;=\;1}^{N-1}\left|x_{i+1}-x_{i}\right|)=log(\sum_{i\;=\;1}^{N-1}\left|\Delta x_{i}\right|) \end{array}$$

where *wl* is the WL feature, |*x*| is the absolute value of *x*, *N* is the number of sample points for a data segment. We extracted *wl* feature from the 5 selected electrodes (the same electrodes as the previous step), and combined the *wl* feature to the *AR* feature. Thus, 30 features were determined for a single trial.

And finally, LDA was used as the classifier.

#### 2.5.2. Method II: dSSA-mulCSP-LDA

Non-stationarity brain sources cause differences between the distributions of electrophysiological signals over time and in particular between the calibration and the application phase (von Bunau et al., 2010), Stationary Subspace Analysis (SSA) (von Bünau et al., 2009) can be used to restrict the decoding to the stationary brain sources. SSA is limited when applying it to multi-class data, and dSSA (Samek et al., 2012; Liu et al., 2015) that trades-off stationarity and discriminativity was used in this study. Common spatial pattern (CSP) (Fukunaga, 1990; Ramoser et al., 2000) has been widely used in BCI literature, mathematically it is realized by simultaneous diagonalization of the covariance matrices for the two classes. For multi-class decoding, CSP with one-to-one strategy has been proved (Liu et al., 2009). In a pre-processing step we apply dSSA to the calibration data and then mulCSP+LDA on the estimated *s*-sources. The details are shown as follows.

SSA (von Bünau et al., 2009) is a novel method to factorize a high-dimensional time-series signal *x*(*t*) into its stationary *S*^*s*^(*t*) and non-stationary *S*^*n*^(*t*) components.

(2) $$\begin{array}{l} x\left(t\right)=AS\left(t\right)=\left[A^{s}\;A^{n}\right]{\left[S^{s}\left(t\right)\;S^{n}\left(t\right)\right]}^{-1} \end{array}$$

where *A* is an invertible matrix. The goal of dSSA is to minimize the distance measured as Kullback-Leibler Divergence *D*_*KL*_, between the distribution of the estimated *s*-sources in each epoch (described by first two moments) and the standard normal distribution. The objective function of dSSA measures the divergence can be written as

(3) $$L(R)=\sum_{i\;=\;1}^{n_{g}}D_{KL}[N({\bar{u}}_{i},{\bar{\sum }}_{i})\|N(\bar{u},\bar{\sum })$$

where *n*_*g*_ is the number of groups, $N({\bar{u}}_{i},{\bar{\sum }}_{i})$ is the average distribution in group *i*, $N\left(ū,\bar{\sum }\right)$ is the average distribution of all groups and *R* is a rotation matrix.

For CSP method, the stationary ECoG signal is represented as *S*_*k*_ with dimensions *ch*×*len*, where *ch* is the number of recording electrodes (i.e., the channel numbers of the signal), and *len* is the number of sample points in one set. The normalized spatial covariance matrix of the ECoG can be obtained from

(4) $$\begin{array}{l} C_{k}=\frac{S_{k}S_{k}^{T}}{trace\left(S_{k}S_{k}^{T}\right)} \end{array}$$

where *k* is the trial number, $S_{k}^{T}$ denotes the transpose of the matrix *S*_*k*_, and $trace\left(S_{k}S_{k}^{T}\right)$ is the sum of the diagonal elements of the matrix $S_{k}S_{k}^{T}$. Let

(5) $$\begin{array}{c} C_{l}=\sum_{k\;\in \;I_{l}}C_{k}\;C_{r}=\sum_{k\;\in \;I_{r}}C_{k} \end{array}$$

where *C*_*l*_, *C*_*r*_ is the average covariance matrix, *I*_*l*_ and *I*_*r*_ are the two index sets of the separate classes (e.g., hand grasp and hand unfold). Set *C* = *C*_*l*_ + *C*_*r*_, the eigenvalue decomposition of *C*

(6) $$\begin{array}{l} C=U_{c}\underset{}{\sum }U_{c}^{T} \end{array}$$

where $\underset{}{\sum }$ is the diagonal matrix, *U*_*c*_ is the eigenvector matrix. The average covariance matrix *C*_*l*_, *C*_*r*_ can be transformed as

(7) $$\begin{array}{cc} S_{l}=PC_{l}P^{T}=U\sum_{l\;\in \;}U^{T};\;S_{r}=PC_{r}P^{T}=U\sum_{r\;\in \;}U^{T} & \;(7) \end{array}$$

where $P=\sqrt{\overset{-1}{\underset{}{\sum }}}U_{c}^{T}$ is the whitening matrix, $\underset{l}{\sum }+\underset{r}{\sum }=I$. The projection matrix *W* could be gained from *W* = *U*^*T*^*P*, the rows of *W* are called spatial filters, and the columns of *W*^−1^ are called spatial patterns. To the *k*-th trial, the filtered signal *Z*_*k*_ = *WS*_*k*_ is uncorrelated. In this work, the log variance of the first *n* rows and last *n* rows (*n* = 3 or 4) of *Z*_*k*_ corresponding to largest *n* eigenvalues and smallest *n* eigenvalues are chosen as feature vectors. One-to-one strategy were employed for three classes decoding in this study, 3 spatial filters were achieved, and finally, obtained 6*n* dimension (i.e., 3^*^2*n*) feature vector for each trial.

Finally, LDA was used as the classifier.

For both method I and method II, a 10 × 10 fold cross validation was adopted to evaluate the classification accuracy among different tasks, which was described as follows: trials were randomly permutated firstly, then equally divided into ten partitions, each partition was used as an unknown test set which was classified by the classifier trained with the remaining nine partitions, and a classification accuracy for each partition was achieved. This process was repeated ten times, and 100 classification accuracy indexes were generated. The average value of these 100 indexes was the final classification accuracy.

## 3. Results

### 3.1. Spatial/spectral selection and analysis

**Figure 4:**
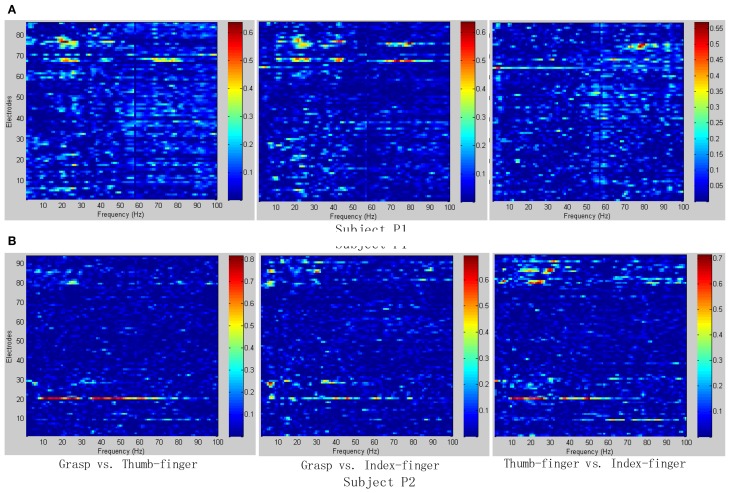
***R*^2^-values for Subject P1 and P2 with two different combinations of classes (Grasp vs. Thumb-finger, Grasp vs. Index-finger, Thumb-finger vs. Index-finger) during seizure monitoring**. The power spectrum shown here was calculated with a frequency bin width of 1 Hz for each electrode. The selected frequency bins of P2 with combination of thumb-finger and index-finger, for example, were 64, 65, 97, 98, 99 within electrodes 3, 4, 9, 10, 16. **(A)** *R*^2^-values of Subject P1; **(B)** *R*^2^-values of Subject P2.

**Figure 5:**
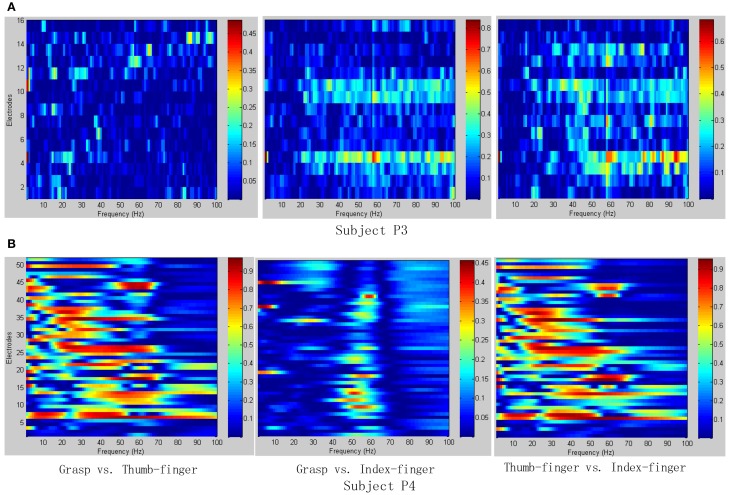
***R*^2^-values for Subject P3 and P4 with two different combinations of classes (Grasp vs. Thumb-finger, Grasp vs. Index-finger, Thumb-finger vs. Index-finger) during awake craniotomy**. The power spectrum shown here was calculated with a frequency bin width of 1 Hz for each electrode. The selected frequency bins of P3 with combination of Grasp and index-finger, for example, were 64, 65, 97, 98, 99 within electrodes 3, 4, 9, 10, 16. And similarly, the selected frequency bins of P3 with combination of thumb-finger and index-finger were 2, 14, 18, 35, 65 within electrodes 4, 11, 12, 13, 14. **(A)** *R*^2^-values of Subject P3; **(B)** *R*^2^-values of Subject P4.

To determine the most discriminative features (electrodes and frequency ranges), *R*^2^-values of the training data set were calculated for all relevant class combinations and for each electrode and each frequency bin. Exemplary *R*^2^-values for two-class combinations with 1 Hz frequency range bins are shown in Figures 4, 5. Subjects P1~P4 were implanted with 87, 95, 16, 52 electrodes, respectively.

As shown in Figure 5A, for example, the selected frequency bins and electrodes of P3 for “Grasp vs. index-finger” and “thumb-finger vs. index-finger” are different, indicating that different types of hand motion is induced by different cortical areas. Figures 4, 5A show the *R*^2^-values of Subject P1, P2, and P3 with two different combinations of classes, the movement-related information is highly localized in a several electrodes, which is in line with previous ECoG studies (Leuthardt et al., 2004). For “Grasp vs. Thumb-finger” and “Thumb-finger vs. Index-finger” of Subject P4 (Figure 5B), the activity in the low frequency band is widespread, and most electrodes over the grid contain movement-related information. For the higher frequency ranges (>80 Hz), the activity is localized, and only a few electrodes contain movement-related information.

**Figure 6:**
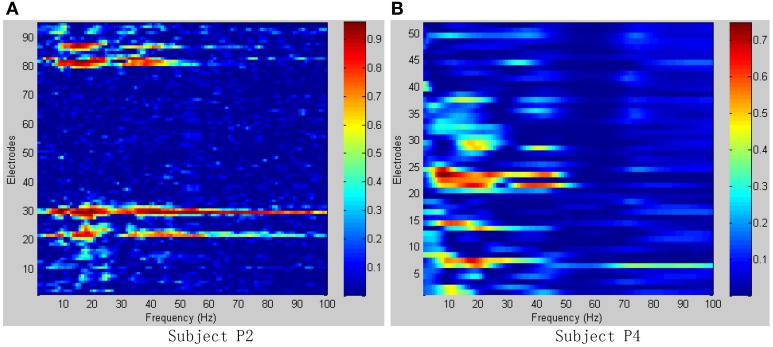
***R*^2^-values for Subject P2, P4 with combinations of motion and idle state**. Integrating all types of hand motions duration as motion state, and all the relax interval as idle state. **(A)** *R*^2^-values of Subject P2; **(B)** *R*^2^-values of Subject P4.

Figure 6 shows the *R*^2^-values for two-class combinations (motion state and idle state) with 1 Hz frequency range bins. For both seizure monitor (P2) and awake craniotomy (P4) signal, the activity in the alpha (8~12 Hz) and beta (13~28 Hz) frequency bands was widespread, and lots of electrodes contained movement-related information. Within the high frequency ranges (>50 Hz), the activity was localized to a few electrodes, and this result was in accordance with the existing literature studies (Spüler et al., 2014).

### 3.2. Decoding of hand motion types

Different class combinations with 10 Hz frequency range bins were tested by cross-validation to estimate how well different hand motions can be decoded. Results from the cross-validation with combination of three and two classes of subjects P1~P4 are presented in Tables 1, 2 with method I and method II, respectively. “Two-class mean” in Tables 1, 2 is the average of all two-class combinations, i.e., average of Thu-Ind, Thu-Gra and Ind-Gra (Thumb, Index, and Grasp are abbreviated with Thu, Ind, and Gra respectively).

**Table 1:** **Three and two classes classification results from the offline analysis applying method I**.

**Sub**.	**Three classes (%)**	**Thumb-index (%)**	**Thumb-grasp mean (%)**	**Index-grasp (%)**	**Two-class**
P1	90.17	97.00	98.75	95.00	96.92
P2	96.00	99.00	95.25	97.00	97.08
P3	91.77	97.50	96.33	88.83	94.22
P4	92.95	81.27	100.00	100.00	93.76

Cross-validation was used to estimate the accuracy.

**Table 2:** **Three and two classes classification results from the offline analysis applying method II**.

**Sub**.	**Three classes (%)**	**Thumb-index (%)**	**Thumb-grasp (%)**	**Index-grasp (%)**	**Two-class mean**
P1	72.00	61.00	86.75	89.00	77.19
P2	93.17	100.00	100.00	88.25	95.35
P3	95.22	95.00	98.50	99.50	97.06
P4	90.36	86.16	100.00	100.00	94.13

Cross-validation was used to estimate the accuracy.

Applying method I, the classification accuracy of three classes for P1~P4 were 90.17, 96.00, 91.77, and 92.95% respectively. And the “two-class mean” of P1~P4 were 96.92, 97.08, 94.22, and 93.76% respectively. For method II, the frequency band of the stationary signal was 50~80 Hz for P1, P3, P4, and 8~25 Hz for P2. The classification accuracy of three classes for P1~P4 were 72.00, 93.17, 95.22, and 90.36% respectively. And the “two-class mean” of P1~P4 were 77.19, 95.35, 97.06, and 94.13% respectively.

**Figure 7:**
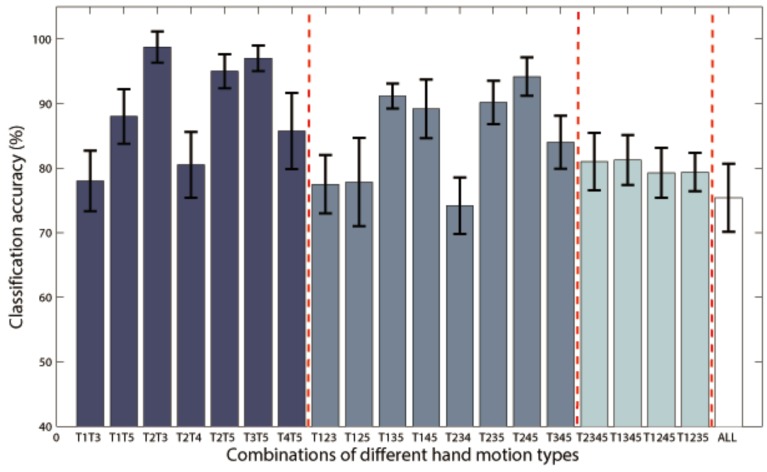
**Classification accuracies of Subject P1 with combinations of 2~5 classes of hand motions**. The power spectrum was calculated with a frequency bin width of 10 Hz for each electrode. Only class combinations with accuracy higher than 70% were presented. T1~5 means hand unfold, hand grasp, thumb-finger motion, wrist motion and index-finger motion respectively. T1T3 is the combination of T1 and T3, and so on for the rest combination. “ALL” is the combination of all the five motion types.

Additional analysis was performed to classify more complex hand motion types. Results of P1 with combinations of 2~5 classes are illustrated in Figure 7, and only class combinations with classification accuracy higher than 70% are presented. The best two-class accuracy was achieved for the combination of grasp and thumb-finger motion with accuracy of 98.75%, the best three-class was combination of grasp, wrist motion and index-finger motion with accuracy of 94.17%, while the best four-class was combination of grasp, thumb-finger motion, wrist motion and index-finger motion with accuracy of 81.25%. The five-class accuracy was acceptable with accuracy of 75.40%.

### 3.3. Comparison of method I and II in awake craniotomy condition

**Figure 8:**
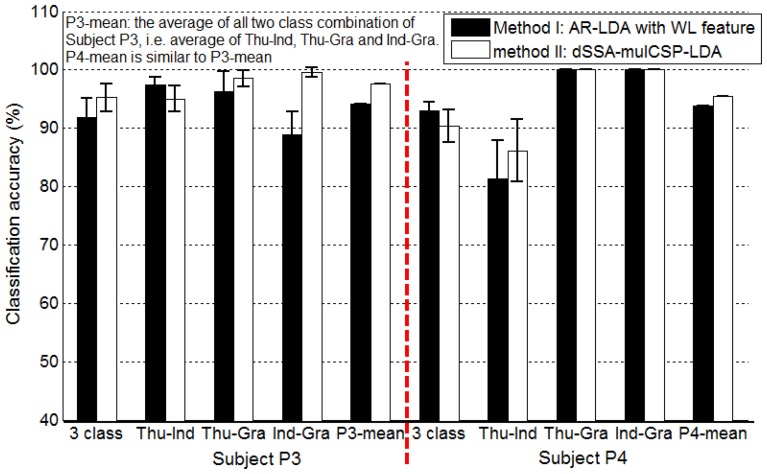
**Classification accuracies of Subject P3, P4 (awake craniotomy) applying method I (AR-LDA with WL feature) and method II (dSSA-mulCSP-LDA)**. “P3-mean” is the average of all two class combination of Subject P3, i.e., average of thu-Ind, Thu-Gra and Ind-Gra. “P4-mean” is similar to “P3-mean.” The power spectrum used in method I was calculated with a frequency bin width of 10 Hz for each electrode. The frequency band of the stationary signal used in method II was 50~80 Hz for P3, P4.

For awake craniotomy signal (P3, P4), method II achieved a better decoding performance than method I, as shown in Figure 8. Particularly, three-class accuracy of P3 was 91.77% with method I and 95.22% with method II, i.e., 3.75% improvement. For Ind-Gra (P3) and Thu-Ind (P4), it had 12.01, 6.01% improvement, respectively. Results indicated that method II was a good algorithm for awake craniotomy signal decoding. Because of the two different clinical conditions (under anesthetic and not), ECoG signals during awake craniotomy and seizure monitoring may have some difference. Subjects was under anesthetic state during awake craniotomy, and both volatile and intravenous anesthetics had deep influence on the central nervous system (Engelhard and Werner, 2006). With increase of end-expiratory concentrations, the spectral edge frequency decreased, the total power and relative power in the delta and theta band increased and the power in the beta band decreased (Schwender et al., 1998; Gugino et al., 2001). These two different clinical conditions may gave a reason to the better decoding performance of method II. And also, the decoding results indicated that method I and II were robust enough for both clinical conditions.

AR-LDA and mulCSP-LDA methods have been widely used in EEG and MEG signal decoding, this study gave an improvement based on the two methods for ECoG signal processing. WL feature was considered in the AR-LDA method, and SSA was applied in the mulCSP-LDA method. Table 3 showed the decoding results of awake craniotomy signal (A1: AR-LDA with WL, A2: traditional AR-LDA; B1: SSA-mulCSP-LDA, B2: traditional mulCSP-LDA). With WL feature, the three-class accuracy of P3 was 91.77%, while 78.67% without WL feature. For Thu-Ind, 5.03 and 3.10% improvements were obtained for P3 and P4. Applying SSA process, the three-class accuracy of P4 was 90.36%, while 86.05% with traditional method. And 3.05% improvement was obtained for P4 with Thu-Ind.

**Table 3:** **Decoding results of awake craniotomy signal applying traditional AR-LDA or CSP-LDA methods, and results using improved methods**.

**Sub**.		**Three classes (%)**	**Thumb-index (%)**	**Thumb-grasp (%)**	**Index-grasp (%)**	**Two-class mean**
P3	A1	91.77	97.50	96.33	88.83	94.22
	A2	78.67	92.83	94.83	87.83	91.83
P4	A1	92.95	81.27	100.00	100.00	93.76
	A2	91.70	78.82	100.00	100.00	92.94
P3	B1	95.22	95.00	98.50	99.50	97.06
	B2	95.33	93.33	98.33	98.50	96.72
P4	B1	90.36	86.16	100.00	100.00	94.13
	B2	86.05	81.52	100.00	100.00	93.84

A1, A2 is the representation of method I with and without Waveform Length feature (WL) respectively. B1, B2 is the representation of method II with and without Stationary Subspace Analysis (SSA) process respectively. Red font indicates results with significant improvement.

## 4. Discussion

Results (Tables 1, 2) obtained by two different methods verified the possibility of decoding multiple hand motion types with high accuracy. Two kinds of subjects were involved in this study, i.e., during awake craniotomy and seizure monitoring processes. Although the clinical condition (Engelhard and Werner, 2006) and signal characteristics (Schwender et al., 1998; Gugino et al., 2001) were different, both methods were robust enough to achieve a high decoding accuracy. Especially, subjects undergoing awake craniotomy achieved a comparable results to the subjects during seizure monitoring. For awake craniotomy subjects P3, P4, the accuracy of three classes were 91.77, 92.95% using method I, and 95.22, 90.36% using method II. It is also important to note that, similar hand motions are considered, like index-finger motion and thumb-finger motion. The accuracies of P1~P4 (index-finger vs. thumb-finger) were 98.75, 99.00 97.50, and 81.27% respectively (Table 1). For P1, the five-class accuracy was 75.40%, which once again showed the possibility of decoding complex hand motions with ECoG signal. These results of the seizure monitor signal were consistence with previous studies (Pistohl et al., 2012; Chestek et al., 2013; Bleichner et al., 2014).

So far, additional important questions need to be answered to accelerate research of ECoG-based BCI, and the ECoG feature selection was one of those (Schalk and Leuthardt, 2011). As AR feature and CSP feature were commonly used for neural electrophysiological signals analysis, two methods, i.e., AR-LDA and mulCSP-LDA, were applied in this studies to further validate the decoding results. Preliminary results indicated that the later one was a good algorithm for awake craniotomy signal decoding. However, this case study mainly focused on the feasibility of decoding awake craniotomy ECoG signal, the best feature and optimal decoding algorithm for intraoperative ECoG signal need to be intensively studied in further researches.

With substantial theoretical and empirical evidence, ECoG-based implant could enhance the functional capability of disabled patients by enabling their ability to modulate their environment, communicate, or control a prosthesis. Looking into the future, as the risk profile is low enough, it may also become reasonable to contemplate implants that augment capabilities in normal functioning adults (Schalk and Leuthardt, 2011). For a pure BCI-purpose implantation, it would be highly necessary to demonstrate reliable neuroprosthetic control during surgical implantation (i.e., during awake craniotomy), in order to verify the placement of electrodes and troubleshoot any technical difficulties during the intraoperation (Fifer et al., 2014). Classifying multiple hand motion types during an awake craniotomy was the crucial first step toward the dexterous neuroprosthetic control during the time of surgical implantation.

For clinical condition, the human ECoG signals were mainly measured on patients with intractable epilepsy and brain tumor. ECoG has been verified as an effective approach for precise identification of eloquent cortex prior to surgical resections adjacent to motor cortex (Roland et al., 2010; Schalk and Leuthardt, 2011; Kamada et al., 2014). Speech and motor cortex could be localized broadly and grossly in real time during an awake craniotomy (Roland et al., 2010). By taking multiple hand motion types into consideration, it has the potential for more precise identification of eloquent cortex, and thus reduces the risk of motor related paralysis after surgical resection.

As the motor imagery is associated with actual movement in ECoG signals (Miller et al., 2010) and it is very hard (and sometimes unable) for patients to execute actual moment during awake craniotomy, ECoG signal may be a good approach for eloquent cortex identification based on motor imagery or intended movement. Using motor imagery ECoG signal during awake craniotomy to localize the eloquent cortex in real time can be a useful BCI system, which may help both patients and neurosurgeons.

ECoG decoding in this case study was completed off-line, so real-time decoding should be performed during an awake craniotomy for optimal placement of electrodes and on-line identification of precise eloquent cortex. In addition, for more precise cortex localization, micro-electrode grids should be taken into consideration, so as to obtain ECoG signal with higher spatial resolution.

## 5. Conclusion

This study verified the possibility of decoding multiple hand motion types during an awake craniotomy, and its accuracy was comparable with subjects during seizure monitoring. Even similar hand motions, like index-finger motion and thumb-finger motion, could be decoded with accuracy of 98.75, 99.00, 97.50, and 81.27% for P1~P4, respectively, which once again showed the possibility of decoding complex hand motions with ECoG signal. Two methods were applied to further validate the decoding results, both of the methods could achieve high decoding accuracy, and method II showed good performance for awake craniotomy signal decoding. We finally suggest that, ECoG signal during awake craniotomy could be used for intraoperative BCI study and precise localization of eloquent cortex, and intensive real-time study should be performed during awake craniotomy.

## Author contributions

DZ and LC conceived the study, DZ and LC designed the experiment, TX, ZW, and LC conducted the experiments. TX, ZW, and DZ analyzed the data. TX, DZ, and XZ co-drafted the paper, XZ coordinated the work.

## Funding

This work is supported by the National Basic Research Program (973 Program) of China (Grant No.2011CB013305, No. 2015CB755500) and the National Natural Science Foundation of China (Grant No.51475292, No.51375296).

### Conflict of interest statement

The authors declare that the research was conducted in the absence of any commercial or financial relationships that could be construed as a potential conflict of interest.
